# Utilization of Glucose-6-Phosphate Dehydrogenase Test and the Prevalence of Enzyme Deficiency in Korea

**DOI:** 10.3390/jcm12093179

**Published:** 2023-04-28

**Authors:** Rihwa Choi, Wonseo Park, Gayoung Chun, Sang Gon Lee, Eun Hee Lee

**Affiliations:** 1 Department of Laboratory Medicine, Green Cross Laboratories, Yongin 16924, Republic of Korea; pirate0720@naver.com; 2 Department of Laboratory Medicine and Genetics, Samsung Medical Center, Sungkyunkwan University School of Medicine, Seoul 06351, Republic of Korea; 3 Infectious Disease Research Center, Green Cross Laboratories, Yongin 16924, Republic of Korea; realdenma@gclabs.co.kr (W.P.); forjund@gclabs.co.kr (G.C.); 4 Green Cross Laboratories, Yongin 16924, Republic of Korea

**Keywords:** hemolytic anemia, glucose-6-phosphate dehydrogenase, utilization, rare disease, Korea

## Abstract

Glucose-5-phosphate dehydrogenase (G6PD) deficiency is an X-linked genetic disorder that affects red blood cells’ metabolism. This retrospective study aimed to investigate the prevalence and characteristics of G6PD testing in Korea. Data were collected from laboratory information systems between July 2021 and June 2022. A total of 5193 patients (1722 males and 3471 females) with a median age of 55.1 years (interquartile range, IQR 44.6 to 64.5) were tested for whole blood G6PD, with 1.6% of tests performed on patients of non-Korean ethnicity. The majority of tests were performed in hospitals (37.7%) or local clinics (34.5%). Interestingly, no female children were tested for whole blood G6PD during the study period. The prevalence of decreased G6PD activity (<7.9 U/g Hb) was 0.4% (19/5111 Koreans and 2/82 non-Koreans), and only seven male patients with G6PD deficiency (<30% of the male median) were identified, with ages ranging from 4.8 months to 50.2 years. No female patients with G6PD deficiency were found. Further research is necessary to determine the clinical significance of G6PD test results and monitor their use.

## 1. Introduction

Glucose 6-phosphate dehydrogenase (G6PD) deficiency is an inherited metabolic disorder that renders red blood cells susceptible to oxidative damage, resulting in hemolytic anemia [1]. This condition is caused by X-linked genetic variants in the G6PD gene [1,2]. Clinical manifestations of G6PD deficiency vary among individuals, ranging from asymptomatic to life-threatening acute hemolytic anemia triggered by exogenous factors such as certain medications, including some antimalarials and antibacterials [1,3,4,5]. Recent advancements in medicine require caution when prescribing drugs that may lead to hemolysis in individuals with G6PD deficiency, such as certain antimalarials, antibiotics, and COVID-19 treatments, including dapsone [1,4,5]. Medical practitioners should consider the possibility of hemolytic anemia, including hyperbilirubinemia in newborns, in patients who display related symptoms and signs, possess a family history of G6PD deficiency, or are from populations with high G6PD deficiency prevalence, such as those of Mediterranean, African, or Southeast Asian descent [1]. The prevalence of G6PD deficiency worldwide varies from 0 to over 30% among ethnic and regional groups [1,6]. The exact prevalence of the G6PD deficiency in Korea is not yet known and varies depending on the population group being studied [7]. For example, among a pediatric hereditary hemolytic anemia cohort including patients newly diagnosed with hereditary hemolytic anemia from 2007 to 2016, the prevalence of G6PD deficiency is estimated to be around 2.4% [7]. Another study performed with blood specimens from Korean soldiers entering training camps reported a 0.0% prevalence of G6PD deficiency [8]. Although G6PD deficiency has been considered a rare disease in Korea, the importance of G6PD testing in various medical conditions has been emphasized due to the increasing number of immigrants and international marriages [1,6,7,8].

In Korea, there are endemic regions of vivax malaria, including northern Gyeonggi-do near the demilitarized zone, where many military personnel are stationed [8,9]. Given that Korea has endemic regions of vivax malaria, G6PD deficiency should be considered an important factor for malaria elimination strategies, particularly as the number of foreign workers and international marriages increases [6,9]. Of particular concern from a public health perspective is the use of 8-aminoquinolines, such as primaquine and tafenoquine, which are the only drugs currently available to treat the hypnozoite stage of Plasmodium vivax infection [5]. G6PD deficiency-related hemolytic anemia has been reported in Korean Army patients who received primaquine administration prior to G6PD deficiency screening [8]. Since 2009, the Korean Disease Control and Prevention Agency (KDCA) has published guidelines for the management of malaria, including information on cautions for anti-malarial drugs in individuals with G6PD deficiency [10]. In 2019, the KDCA launched the “Five-Year Action Plan for Malaria” (2019–2023) [9]. More recently, the KDCA has included information on rapid diagnostic tests for G6PD deficiency [10].

The analytical methods for G6PD activity tests are not standardized, and results of G6PD activity tests should be interpreted along with the adjusted male median [2,5,11]. The World Health Organization (WHO) has published guidelines for technical consultation series (TSS) for evaluating diagnostic tests for G6PD activity that categorize G6PD activity as deficient when it is less than 30% of the adjusted male median and intermediate in females when it is 30–80% of the adjusted male median [2,5,10]. For example, a previous study performed in Korea describing analytical methods using point-of-care tests reported a male median value of 7.582 U/g Hb [8]. Other studies performed in Korea using a kinetic ultraviolet assay with a whole blood kit reported a male median value of 11.5 U/g Hb [6,12]. The 2022 WHO report updated the classification of G6PD variants based on G6PD activity, with variants having activity below 20% of the adjusted male median classified as “class A” for chronic non-spherocytic hemolytic anemia, below 45% as “class B” for triggered acute hemolytic anemia, between 60 and 150% as “class C” with no hemolysis, and the remaining variants classified as “class U” with uncertain clinical significance [5]. 

However, there has been a lack of information regarding the utilization of G6PD assay tests and the results of G6PD activity tests based on various factors such as age, ethnicity, medical institution, geographic region, and adherence to the biochemical criteria for the G6PD assay, as per the WHO guidelines, in a large Korean population. Therefore, the aim of this study was to evaluate the prevalence of G6PD deficiency in Korea by analyzing the utilization of G6PD activity tests and results from a large number of patients using the current WHO classifications for G6PD enzyme activity. Green Cross Laboratories is one of the largest referral clinical laboratories in Korea, performing G6PD activity tests. As there has been a lack of studies on G6PD deficiency in a large number of patients in Korea, this study provides important basic information about G6PD deficiency in Korea and the clinical management of individuals with G6PD deficiency.

## 2. Materials and Methods

### 2.1. Study Subjects

In this retrospective study, we analyzed data obtained from Green Cross Laboratories’ laboratory information system between 1 July 2021 and 30 June 2022, to investigate the prevalence of decreased glucose-6-phosphate dehydrogenase (G6PD) in patients who underwent whole blood G6PD testing. 

Among the 5939 G6PD test results collected, we excluded test results for patients with missing age or sex data (*n* = 456). Out of the remaining 5483 results, we also excluded data from repeatedly measured tests on the same individuals (135 patients measured twice, 35 patients measured 3 times, 13 patients measured 4 times, 6 patients measured 5 times, 3 patients measured 6 times, and 1 patient measured 8 times during the study period), and only their initial measurements were included for further analysis. Therefore, a total of 5193 G6PD test results for 5193 subjects were included in the final analysis.

### 2.2. Definitions

To ensure patient privacy, all data were anonymized before statistical analysis. Patient age was categorized into 10-year age groups (<10 years, 10–19 years, 20–29 years, 30–39 years, 40–49 years, 50–59 years, 60–69 years, 70–79 years, and ≥80 years). Ethnicities were classified as Korean and non-Korean in the laboratory information system.

We defined decreased G6PD activity as a test result < 7.9 U/g Hb based on verification using the laboratory’s clinical specimens and the reference interval suggested by the manufacturer’s information [13]. In addition, we categorized G6PD activity based on the percentage of the adjusted male median according to the WHO TSS (“deficient” when activity was <30% of the adjusted male median and “intermediate” in females when G6PD activity was 30–80% of the adjusted male median) [2,5,6,11]. The adjusted male median was defined as the median G6PD activity of all male participants after excluding samples with less than 10% of the overall median activity [6,12]. We also classified G6PD enzyme activity according to the WHO classification of G6PD variants in homozygous and hemizygous individuals (<20% for class A, 20 to <45% for class B, 60–150% for class C, and others for class U) to assess the possible prevalence of G6PD deficiency with various types of G6PD variants [5]. 

Medical institutions were categorized into local clinics, hospitals, university hospitals, other referral clinical laboratories, public medical centers, and armed forces hospitals.

There are 17 administrative districts in Korea: Seoul, Gyeonggi-do, Gangwon-do, Chungcheongbuk-do, Chungcheongnam-do, Jeollabuk-do, Jeollanam-do, Gyeonsangbuk-do, Gyeonsangnam-do, Busan, Daegu, Incheon, Gwangju, Daejeon, Ulsan, Sejong City, and Jeju-do (Jeju Island). To investigate the differences in G6PD test utilization and the prevalence of G6PD deficiency, we grouped the administrative districts of Korea into six categories based on geographic proximity and the characteristics of G6PD deficiency as a rare disease: (1) Seoul and Gyeonggi-do (Seoul, Gyeonggi-do, and Incheon); (2) Gang-won-do; (3) Chungcheong province (Chungcheongbuk-do, Chungcheongnam-do, Daejeon, and Sejong City); (4) Jeolla province (Jeollabuk-do, Jeollanam-do, Gwangju); (5) Gyeonsang province (Gyeonsangbuk-do, Gyeonsangnam-do, Busan, Daegu, and Ulsan); and (6) Jeju-do (Jeju Island).

To estimate the number of patients who underwent G6PD testing in Korea, we indirectly assessed the number of patients using the reimbursable test code D0550 for the erythrocyte enzyme assay from the Electronic Data Interchange code assigned by the Health Insurance Review and Assessment Service (HIRA) Healthcare Bigdata Hub [14]. We also assessed the number of patients managed for G6PD deficiency anemia using the 10th Revision, Clinical Modification of the International Statistical Classification of Diseases and Related Health Problems (ICD-10-CM) D550 for G6PD deficiency anemia in the HIRA Bigdata Hub [15]. In the database maintained by HIRA, administrative districts in Korea were classified as in the original classification.

### 2.3. Analytical Methods

The G6PD level in whole blood was measured using G6PDH kits (Ben Srl Biochemical Enterprise, Milan, Italy) that used a spectrophotometric assay on a URIT-880 analyzer (URIT, Shenzen, China) to measure the formation of nicotinamide adenine dinucleotide phosphate molecules following the manufacturer’s instructions. The manufacturer’s reference interval for G6PD activity at 37 °C ranged from 7.9 to 16.3 U/g Hb. The accuracy of the whole blood G6PD assay was confirmed by a proficiency testing program that included a G6PDS survey and accreditation by the College of American Pathologists. 

### 2.4. Statistical Analysis

Non-normally distributed quantitative values were presented as medians and interquartile ranges, while qualitative values were presented as numbers and percentages. The prevalence of decreased G6PD activity (<7.9 U/g Hb), G6PD deficiency by WHO TSS classification, possible G6PD deficiency with G6PD variants according to WHO classification, sex, age group, ethnicity, and type of medical institution were compared using chi-square tests. To address the potential source of bias, the utilization of the G6PD test was assessed along with data from the public database of the HIRA [14,15]. A *p*-value of less than 0.05 was considered statistically significant using the MedCalc statistical software version 20.216 (MedCalc Software Ltd., Ostend, Belgium; https://www.medcalc.org; accessed on 28 February 2023). Maps for G6PD test utilization and the prevalence of G6PD deficiency were created using R software (version 4.2.2; http://www.R-project.org/; accessed on 10 March 2023).

## 3. Results

### 3.1. Baseline Characteristics of Study Subjects

During the study period, 5193 patients (1722 males and 3471 females) with a median age of 55.1 years (interquartile range: 44.6 to 64.5) underwent whole blood G6PD testing, of whom 1.6% were of ethnicities other than Korean, as summarized in Table 1. G6PD testing was performed more frequently in females than males in both Koreans (66.9% female) and other ethnicities (64.6% female), with no significant difference in the sex distribution between Koreans and other ethnicities. The majority of patients belonged to the age groups of 50 to 59 years and 60 to 69 years (26.5% and 23.3%, respectively), and most of them visited hospitals (37.7%), followed by local clinics (34.5%). During the study period, no female children underwent whole-blood G6PD testing. The largest proportion of patients was from Seoul and Gyeonggi-do (68.8%), and the male-to-female ratio was almost 1:1 in Jeolla province. However, specimens from Jeju Island (Jeju-do) were not requested for G6PD testing during the study period in any of the administrative districts.

**Figure 1:**
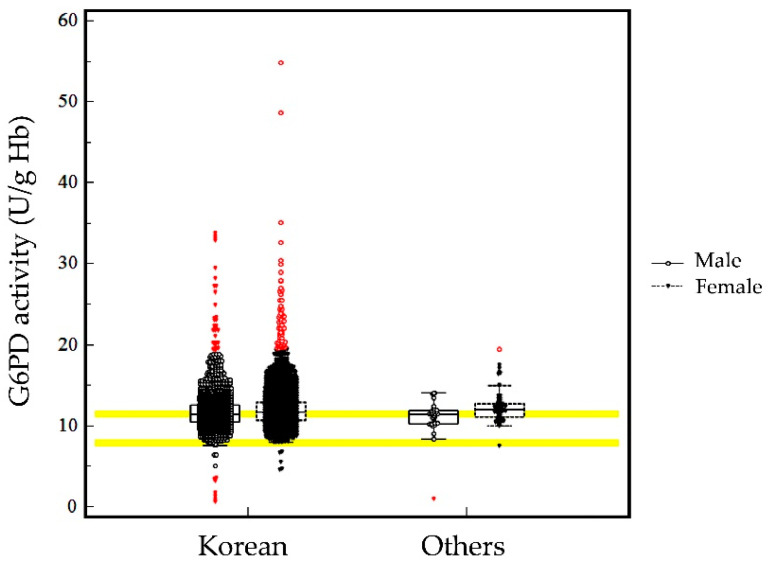
G6PD enzyme activity. Black open circles represent data from male subjects, and black closed inverted triangles represent data from female subjects. Boxes represent interquartile ranges; horizontal lines in the box at the medians; inner fences represent 3 × interquartile levels; and red dots (inverted red triangles for males and a red open circle for females) represent value outside the fences in the box-and-whisker plot. The upper bold yellow line is the adjusted male median (11.4 U/g Hb), and the lower bold yellow line is the lower limit of reference intervals (7.9 U/g Hb).

The median G6PD activity for the entire study population was 11.6 U/g Hb. The adjusted male median was calculated by excluding results with less than 10% of the overall median activity from all male participants, which was 11.4 U/g Hb. The distribution of G6PD activity is presented in Figure 1. There was no significant difference in G6PD activity between ethnicities (Korean vs. non-Korean, *p* > 0.05); however, there was a significant difference in G6PD activity between males and females (*p* < 0.0001).

### 3.2. The Prevalence of Decrased G6PD Enzyme Activity

The prevalence of decreased G6PD (<7.9 U/g Hb) was 0.4% (19/5111 Korean and 2/82 non-Korean), with a median G6PD level of 4.4 U/g Hb (IQR 1.6 to 6.7). The overall prevalence of G6PD deficiency ranged from 0.2% to 0.4%, depending on the classification criteria used. 

The prevalence of decreased G6PD activity (<7.9 U/g Hb), G6PD deficiency by WHO TSS classification (<30% of adjusted male median), and possible G6PD variant prevalence were significantly different between sexes (*p* = 0.0009, <0.0001, and 0.0015, respectively). The overall prevalence of G6PD deficiency by each classification criterion was significantly different between sexes in Koreans but not in other ethnicities (n = 82, *p* > 0.05). The prevalence of G6PD deficiency by age, sex, ethnicity, type of medical institution, and geographic region according to different criteria is summarized in Table 2. 

There were seven patients with G6PD deficiency (<30% of the male median), and they were all male. Among them, six were Korean. None of the female patients were classified as having G6PD deficiency (<30% of the male median). The male-to-female ratio of patients with decreased G6PD (<7.9 U/g Hb) was 2.8 to 1 in Koreans. The overall prevalence of females with intermediate G6PD activity (30–80% of the male median) was 2.9%.

G6PD deficiency and intermediate G6PD were widely distributed across age groups. The highest prevalence of decreased G6PD (<7.9 U/g Hb) and G6PD deficiency (<30% of male median) was observed in the pediatric population with ages ranging from 0 to 19 years, and the rates were 3.7% and 1.6%, respectively. The oldest patient with G6PD deficiency (<30%) was a 50.2-year-old man, and the youngest was a 4.8-month-old boy. Specimens of patients with G6PD deficiency were requested from university hospitals (n = 5) and hospitals (n = 2). Specimens requested from university hospitals had more abnormal G6PD results to various extents than those from other types of medical institutions (*p* < 0.05). The geographic regions of six male patients with G6PD deficiency were Seoul and Gyeonggi-do (n = 4) and Jeolla province (n = 2). The geographic region for one foreign male subject with G6PD deficiency was Chungcheong Province. 

**Figure 2:**
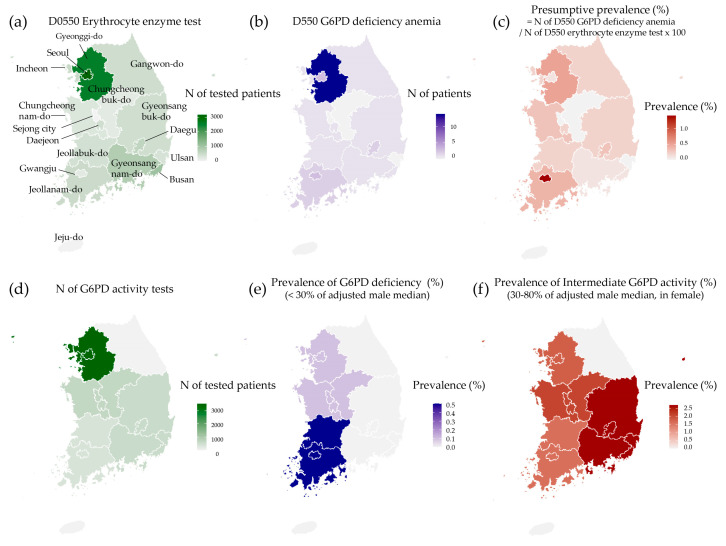
Number and prevalence of patients with G6PD deficiency among geographic regions. (**a**) The number of patients tested for erythrocyte enzyme activity test, available from the public database by HIRA; (**b**) the number of patients managed for G6PD deficiency in 2021 (available from the public database by HIRA); (**c**) the presumptive prevalence of G6PD deficiency (%) based on the number of G6PD deficiency patients/the number of patients tested for erythrocyte enzyme activity test × 100; (**d**) the number of patients tested for G6PD activity in the study population; (**e**) the prevalence of patients with G6PD deficiency (<30% of adjusted male median) in the study population; (**f**) the prevalence of patients with intermediate G6PD activity in female subjects (30–80% of adjusted male median) in the study population.

The total number of subjects tested for G6PD activity, the number of those with G6PD deficiency by different criteria in the present study, the number of patients managed for G6PD deficiency anemia, and the number of patients tested for erythrocyte enzyme tests in the public database by HIRA are summarized in Figure 2 by geographic region.

## 4. Discussion

In the present study, we investigated the recent information on the utilization of the G6PD test and the prevalence of decreased G6PD activity according to different criteria for assessing G6PD deficiency. 

The results of this study were comparable to previous studies performed in Korea. The prevalence of decreased G6PD activity was similar to that reported in previous studies conducted in Korea [6,7,8]. In this study, the male median value was 11.4 U/g Hb, which was also comparable to previous studies in Korea that used the same kinetic UV assay using a whole blood kit (11.5 U/g Hb) [5,12].

In the present study population, G6PD testing was more frequent among females than males. Although Green Cross Laboratories is a referral laboratory and the clinical information for the use of the G6PD test is limited, the higher utilization of G6PD testing in females might reflect the higher prevalence of anemia in females compared to males and the use of G6PD testing in the context of anemia diagnosis and management [1,15,16]. However, no female subjects were identified as having G6PD deficiency (females represented <30% of the male median) in this study population. The male-to-female ratio of 2.8 to 1 in Korean patients with decreased G6PD (<7.9 U/g Hb) was comparable to data from the public database by HIRA, in which the male-to-female ratio was approximately 3.3 to 1 among patients with G6PD deficiency in 2021, regardless of initial visits or follow-up [15]. Because of the small number of patients who were not Korean, calculating the ratio with statistical significance was not possible. The ratio observed in this study aligned with the current knowledge of the disease characteristics of X-linked genetic polymorphisms [1]. Although genetic tests for the G6PD gene are important for the diagnosis of G6PD deficiency, G6PD enzyme tests to predict the pathogenicity of variants have become popular and are considered the best tests due to their potential to cause clinical manifestation [1,2]. 

In this study, the G6PD enzyme activity test was underutilized on Jeju Island (Jeju-do). This result was comparable with those observed in the public database by HIRA. In the database, only one patient was tested for erythrocyte activity from Jeju Island in the review year of 2021 [14]. This underutilization may be due to the small number of residents (the population was only 1.3% of Seoul and Gyeonggi-do in 2021) and/or the differences in the subject characteristics, such as healthcare behaviors, geography, culture, and access to healthcare facilities on Jeju Island [17]. 

In the present study, the prevalence of G6PD was highest among the pediatric population, and this prevalence was comparable to previous findings in the Korean pediatric population [7]. Furthermore, G6PD deficiency was distributed throughout all age groups. This finding was also comparable to previous studies showing that most subjects are asymptomatic, which highlights the importance of clinicians’ awareness of G6PD deficiency for the diagnosis and management of patients [1,18,19,20]. It is important to note that G6PD deficiency is relatively rare in Korea compared with some other countries in Asia and the Middle East [1,6,7]. Nonetheless, healthcare providers in Korea should still be aware of the condition, as it can have important implications for the management of certain medical conditions and the use of certain medications [1,6,7]. 

Korean clinical practice guidelines for the diagnosis of hereditary hemolytic anemia released in June 2022 included the G6PD enzyme activity tests because individuals with genetic variants of the G6PD gene are mostly asymptomatic throughout their lifetime; however, these patients may develop acute and very severe hemolytic anemia [1,18]. Although the 2022 Korean clinical practice guidelines for the diagnosis of hereditary hemolytic anemia mentioned erythrocyte enzyme tests using the tandem mass spectrometry method, this test is not reimbursed by HIRA or clinically available in Korea [18]. Under the reimbursement system of HIRA, only erythrocyte enzyme tests using spectrophotometric methods are reimbursable. According to the database maintained by HIRA, the G6PD activity test and the pyruvate kinase test are grouped under the same code of D0550 and are named the erythrocyte enzyme test [14]. Therefore, the exact use of the G6PD activity test is not available. The strength of this study was the large number of G6PD activity tests performed during the study period, which represented data from throughout Korea. According to the HIRA database, in the review year 2021, 12,323 patients were tested with the erythrocyte enzyme test, and the testing number in our study—5111 Korean subjects—represented about 41.5% of them. G6PD deficiency is regarded as a rare disease in Korea, and the assays for G6PD activity have been performed in referral laboratories specialized for those tests. Therefore, the present study seems representative of G6PD test use in Korea.

The limitations of this study include the lack of clinical information associated with hemolytic anemia, causes for G6PD testing, and other triggering factors such as clinical symptoms and signs, comorbidities, and medications. The information on the confirmation of G6PD deficiencies by molecular tests and their relationship with malaria management in the presence of different molecular variants is also limited. Various molecular variants of the G6PD gene can result in diverse biochemical phenotypes, and the identification of G6PD enzyme deficiency remains the most reliable method to determine enzyme-deficient variants that may potentially lead to clinical manifestations [1,4,5]. The information regarding the presence of conditions such as thalassemia (which is another rare disease in Korea), anemia, and a high reticulocyte count, which may have affected the G6PD test results, as well as whether the parents of pediatric patients underwent screening, was also limited [19]. The study period included the COVID-19 pandemic, during which the use of G6PD activity tests may have been different from non-pandemic situations [21,22,23]. The low number of G6PD patients may have influenced the prevalence. 

However, this study is novel in that it provides information on the test utilization of the G6PD assay and the results of the G6PD test according to age, ethnicity, medical institution, geographic regions, and the use of criteria for the biochemical G6PD assay based on WHO criteria in a large Korean population. The large population in the present study provides representative data for G6PD activity testing in Korea and helps to understand the current use status and disease burden of G6PD deficiency in Korea. As Korean clinical practice guidelines for management of hereditary hemolytic anemia and malaria have recently been introduced and include the implication of G6PD deficiency and diagnostic tests for G6PD, future studies, including detailed clinical histories and G6PD genetic studies, are needed to clarify the clinical implication of this test in strategic information for public health in Korea.

## 5. Conclusions

In conclusion, in this study, we investigated the use of G6PD activity tests and the prevalence of decreased G6PD activity using different classification criteria in a large patient population in Korea. The overall prevalence of patients with decreased G6PD activity was 0.4%, and no female subjects were identified during the study period. The prevalence of G6PD deficiency was highest in the pediatric population, but it was also observed in the elderly. The G6PD activity test was found to be underused in female children and individuals residing on Jeju Island. Considering the 2022 Korean guidelines for hemolytic anemia and malaria management, which emphasize the importance of G6PD tests, this study provides basic information for the transition period of clinical guidelines assessing G6PD deficiency anemia for strategic public health programs in Korea. Future studies on the impact of comprehensive approaches, including genetic tests, on clinical outcomes are needed.

## Figures and Tables

**Table 1:** Baseline characteristics of the study population.

Characteristics	Characteristics	Total (*n* = 5193)	Male (*n* = 1772)	Female (*n* = 3471)
Age, year (median, IQR)	Age, year (median, IQR)	55.1 (44.6 to 64.5)	57.8 (43.2 to 66.7)	53.8 (44.9 to 63.1)
	0 to 9 years	157 (3.0%)	107 (6.2%)	50 (14.4%)
	10 to 19 years	110 (2.1%)	67 (3.9%)	43 (1.2%)
	20 to 29 years	219 (4.2%)	91 (5.3%)	128 (3.7%)
	30 to 39 years	424 (8.2%)	110 (6.4%)	314 (9.0%)
	40 to 49 years	968 (18.6%)	212 (12.3%)	756 (21.8%)
	50 to 59 years	1375 (26.5%)	364 (21.1%)	1011 (29.1%)
	60 to 69 years	1208 (23.3%)	469 (27.2%)	739 (21.3%)
	70 to 79 years	508 (9.8%)	227 (13.2%)	281 (8.1%)
	≥80 years	224 (4.3%)	75 (4.4%)	149 (4.3%)
Ethnicity	Korean	5111 (98.4%)	1693 (33.1%)	3418 (66.9%)
	Non-Korean	82 (1.6%)	29 (35.4%)	53 (64.6%)
Medical institution	Local clinics	1792 (34.5%)	693 (40.2%)	1099 (31.7%)
	Hospitals	1959 (37.7%)	515 (29.9%)	1444 (41.6%)
	University hospitals	625 (12.0%)	312 (18.1%)	313 (9.0%)
	Other referral clinical laboratories	635 (12.2%)	144 (8.4%)	491 (14.1%)
	Public medical centers	173 (3.%)	49 (2.8%)	124 (3.6%)
	Armed forced hospitals	9 (0.2%)	9 (0.5%)	0 (0.0%)
Geographic region	Seoul and Gyeonggi-do	3532 (68.8%)	1157 (67.2%)	2375 (68.4%)
	Chungcheong province	677 (13.0%)	205(11.9%)	472 (13.6%)
	Gyeongsang province	590 (11.4%)	169 (9.8%)	421 (12.1%)
	Jeolla province	384 (7.4%)	185 (10.7%)	199 (5.7%)
	Gangwon-do	10 (0.2%)	6 (0.3%)	4 (0.1%)
	Jeju-do	0 (0.0%)	0 (0.0%)	0 (0.0%)
G6PD activity	G6PD, U/g Hb (median, IQR)	11.6 (10.7 to 12.8)	11.4 (10.5 to 12.6)	11.7 (10.8 to 12.9)
	G6PD, <7.9 U/g Hb (n, %)	21 (0.4%)	15 (0.9%)	6 (0.2%)
G6PD deficiency by WHO TSS	<30% of the adjusted male median	7 (0.4%)	7 (0.4%)	0 (0.0%)
	Intermediate G6PD in female (30–80%)	101 (2.9%)	Not applicable	101 (2.9%)
Possible G6PD variant classification	Class A (<20%, chronic non-spherocytic hemolytic anemia)	6 (0.1%)	6 (0.3%)	0 (0.0%)
	Class B (<45%, acute hemolytic anemia)	6 (0.1%)	4 (0.2%)	2 (0.1%)
	Class C (60–150%, no hemolysis)	5003 (96.3%)	1650 (95.8%)	3353 (96.6%)
	Class U (others, uncertain clinical significance)	178 (3.4%)	62 (3.6%)	116 (3.3%)

Abbreviations: Hb—hemoglobin; IQR—interquartile range; G6PD—whole blood glucose-6-phosphatase dehydrogenase; WHO TSS—World Health Organization technical consultation series for evaluating diagnostic tests for G6PD activity. Intermediate classification is only applicable for females according to the WHO TSS for G6PD activity classification.

**Table 2:** Prevalence of decreased G6PD enzyme activity with age by different classifications.

Characteristics	Total *n*	Decreased G6PD (<7.9 U/g Hb)	G6PD Deficiency (<30%)	Intermediate G6PD in Female * (30–80%)	Class A (<25%, Chronic Non-Spherocytic Hemolytic Anemia)	Class B (<45%, Triggered Acute Hemolytic Anemia)	Class U (45–60% or >150%, Uncertain Clinical Significance)
0 to 9 years	157 (3.0%)	5 (3.2%)	3 (1.9%)	4 (8.0%)	3 (1.9%)	2 (1.3%)	20 (12.7%)
10 to 19 years	110 (2.1%)	5 (4.5%)	1 (0.9%)	1 (2.3%)	1 (0.9%)	0 (0.0%)	13 (11.8%)
20 to 29 years	219 (4.2%)	1 (0.5%)	1 (0.5%)	4 (3.1%)	0 (0.0%)	1 (0.5%)	7 (3.2%)
30 to 39 years	424 (8.2%)	1 (0.2%)	1 (0.2%)	11 (3.5%)	1 (0.2%)	0 (0.0%)	10 (2.4%)
40 to 49 years	968 (18.6%)	0 (0.0%)	0 (0.0%)	14 (1.9%)	0 (0.0%)	1 (0.0%)	39 (4.0%)
50 to 59 years	1375 (26.5%)	3 (0.2%)	1 (0.1%)	31 (3.1%)	1 (0.1%)	2 (0.0%)	29 (2.1%)
60 to 69 years	1208 (23.3%)	2 (0.2%)	0 (0.0%)	30 (4.1%)	0 (0.0%)	1 (0.1%)	36 (3.0%)
70 to 79 years	508 (9.8%)	3 (0.6%)	1 (0.0%)	5 (1.8%)	0 (0.0%)	1 (0.2%)	15 (3.0%)
≥80 years	224 (4.3%)	1 (0.4%)	2 (0.0%)	1 (0.7%)	0 (0.0%)	1 (0.4%)	9 (4.0%)
Korean	5111 (98.4%)	19 (0.4%)	6 (0.1%)	Not applicable	5 (0.1%)	4 (0.1%)	62 (3.4%)
Male	1693 (33.1%)	14 (0.8%)	6 (0.4%)	Not applicable	5 (0.3%)	4 (0.2%)	62 (3.7%)
Female	3418 (66.9%)	5 (0.1%)	0 (0.0%)	100 (2.9%)	0 (0.0%)	2 (0.1%)	114 (3.3%)
Non-Korean	82 (1.6%)	2 (2.4%)	1 (1.2%)	Not applicable	1 (1.2%)	0 (0.0%)	2 (2.4%)
Male	29 (35.4%)	1 (3.4%)	1 (3.4%)	Not applicable	1 (3.4%)	0 (0.0%)	0 (0.0%)
Female	53 (64.6%)	1 (1.9%)	0 (0.0%)	1 (1.9%)	0 (0.0%)	0 (0.0%)	2 (3.8%)
Local clinics	1792 (34.5%)	2 (0.1%)	0 (0.0%)	37 (2.1%)	0 (0.0%)	0 (0.0%)	22 (1.2%)
Hospitals	1959 (37.7%)	7 (0.4%)	2 (0.1%)	34 (1.7%)	1 (0.1%)	4 (0.2%)	80 (4.1%)
University hospitals	625 (12.0%)	12 (1.9%)	5 (0.8%)	22 (3.5%)	5 (0.8%)	2 (0.3%)	54 (8.6%)
Other referral clinical laboratories	635 (12.2%)	0 (0.0%)	0 (0.0%)	6 (0.9%)	0 (0.0%)	0 (0.0%)	18 (2.8%)
Public medical centers	173 (3.%)	0 (0.0%)	0 (0.0%)	2 (1.2%)	0 (0.0%)	0 (0.0%)	4 (2.2%)
Armed forced hospitals	9 (0.2%)	0 (0.0%)	0 (0.0%)	0 (0.0%)	0 (0.0%)	0 (0.0%)	0 (0.0%)

* Intermediate classification is only applicable for females in the World Health Organization technical consultation series for evaluating diagnostic tests for G6PD activity classification.

## Data Availability

The datasets generated and analyzed during the current study are available from the corresponding authors on reasonable request.
